# Prognostic nomogram for previously untreated adult patients with acute myeloid leukemia

**DOI:** 10.18632/oncotarget.12245

**Published:** 2016-09-26

**Authors:** Zhuojun Zheng, Xiaodong Li, Yuandong Zhu, Weiying Gu, Xiaobao Xie, Jingting Jiang

**Affiliations:** ^1^ Department of Hematology, The Third Affiliated Hospital of Soochow University, China; ^2^ Department of Tumor Biological Treatment, The Third Affiliated Hospital of Soochow University, China; ^3^ Cancer Immunotherapy Engineering Research Center of Jiangsu Province, China; ^4^ Institute of Cell Therapy, Soochow University, China; ^5^ Department of Oncology, The Third Affiliated Hospital of Soochow University, China

**Keywords:** acute myeloid leukemia, nomogram, prognosis, prediction, TCGA

## Abstract

This study was designed to perform an acceptable prognostic nomogram for acute myeloid leukemia. The clinical data from 311 patients from our institution and 165 patients generated with Cancer Genome Atlas Research Network were reviewed. A prognostic nomogram was designed according to the Cox's proportional hazard model to predict overall survival (OS). To compare the capacity of the nomogram with that of the current prognostic system, the concordance index (C-index) was used to validate the accuracy as well as the calibration curve. The nomogram included 6 valuable variables: age, risk stratifications based on cytogenetic abnormalities, status of *FLT3*-ITD mutation, status of *NPM1* mutation, expression of CD34, and expression of HLA-DR. The C-indexes were 0.71 and 0.68 in the primary and validation cohort respectively, which were superior to the predictive capacity of the current prognostic systems in both cohorts. The nomogram allowed both patients with acute myeloid leukemia and physicians to make prediction of OS individually prior to treatment.

## INTRODUCTION

Acute myeloid leukemia (AML) is a highly aggressive hematological malignancy [1]. The clinical outcome of patients with AML relates with morphology, immunophenotype, molecular and cytogenetic abnormalities of disease [2–4]; and patient factors including age at diagnosis, race-ethnicity, and socio-economic status [5].

In the last a few decades, the influences of recurrent cytogenetic and molecular abnormalities have been developed. Mutations in genes *nucleophosmin 1* (*NPM1*) [6] and *fms-related tyrosine kinase 3* internal tandem duplications (*FLT3*-ITD) [7] can accurately improve the survival of the patients with cytogenetically normal AML.

A few groups have intended to build prognostic models by integrating primary disease-related factors and patient factors to establish prognostic systems [8]. However, few of them were acknowledged commonly for that the data of them were obtained from different patient series and included heterogeneous treatments. Nowadays, the National Comprehensive Cancer Network (NCCN) Clinical Practice Guidelines in Oncology: Acute Myeloid Leukemia has been acknowledged as the best prognostic and predictive system in the world [9] However, there are still limitations for all patients with AML.

Various nomograms have been validated in studies of several malignancies. They provide better statistical predictive models [10, 11]. The visual format of nomogram helps the patients and their physicians understand their prognoses. In this study, we sought to design a reliable predictive nomogram for predicting the survival for both patients and clinicians. Forecasting ability of this nomogram was validated in a completely independent cohort.

## RESULTS

### Patient characteristics

The baseline clinical characteristics of all patients are listed in Table 1. There were 476 patients diagnosed as AML included in this study with 10 months of median time of follow-up. The median age was 57 years (range, 18–88 years). The proportions of the advanced age stratification were 53.8% (< 60 years), 35.3% (60−75 years) and 10.9% (> 75 years). Most of the patients (*n* = 312, 65.5%) were stratified as intermediate risk based on cytogenetic abnormalities; while the numbers of patients stratified as favorable and poor risk were 54 (11.3%) and 110 (23.2%), respectively. *FLT3*-ITD and *NMP1* mutations occurred in 35.5% and 33.6% of patients, respectively. Of all the patients, 84.7% were positive for myeloperoxidase (MPO), 78.8% were positive for cluster of differentiation 34 (CD34), 66.8% were positive for CD56, and 90.3% were positive for human leukocyte antigen DR (HLA-DR) in immunophenotype. Similar and different clinical characteristics could be found in all cohorts (Table 1). The 1-year, 2-year and 3-year overall survival rates were 57.6%, 34.8%, 25.1% for the primary cohort and 55.3%, 34.0%, 22.9% for the validation cohort, respectively.

**Table 1:** Clinical characteristics of patients with acute myeloid leukemia

Characteristic	All patients *No*. (%)	Primary cohort *No*. (%)	Validation cohort *No*. (%)	*P*-value
Total	476	311	165	
Sex				
Male	262 (55.0)	171 (55.0)	91 (55.2)	0.972
Female	214 (45.0)	140 (45.0)	74 (44.8)	
Age (years)				
< 60	256 (53.8)	172 (55.3)	84 (50.9)	0.682
60–75	168 (35.3)	108 (34.7)	60 (36.4)	
> 75	52 (10.9)	31 (10.0)	21 (12.7)	
CA				
Favorable-risk	54 (11.3)	34 (10.9)	20 (12.1)	0.891
Intermediate-risk	312 (65.5)	206 (66.3)	106 (64.2)	
Poor-risk	110 (23.2)	71 (22.8)	39 (23.6)	
*FLT3*-ITD				
Presence	169 (35.5)	126 (40.5)	43 (26.1)	0.001
Absence	307 (64.5)	185 (59.5)	122 (73.9)	
*NPM1* mutation				
Presence	160 (33.6)	116 (37.3)	44 (26.7)	0.019
Absence	316 (66.4)	195 (62.7)	121 (73.3)	
Immunophenotype				
MPO				
Positive	403 (84.7)	266 (85.5)	137 (83.0)	0.471
Negative	73 (15.3)	45 (14.5)	28 (17.0)	
CD34				
Positive	375 (78.8)	239 (76.8)	136 (82.4)	0.157
Negative	101 (22.2)	72 (23.2)	29 (17.6)	
CD56				
Positive	318 (66.8)	199 (64.0)	119 (72.1)	0.073
Negative	158 (33.2)	112 (36.0)	46 (27.9)	
HLA-DR				
Positive	430 (90.3)	278 (89.4)	152 (92.1)	0.347
Negative	46 (9.7)	33 (10.6)	13 (7.9)	

Abbreviations: CA, cytogenetic abnormalities; *FLT3*-ITD, *fms-related tyrosine kinase 3* internal tandem duplications; *NPM1, nucleophosmin 1*; MPO, myeloperoxidase; HLA-DR, human leukocyte antigen DR.

### Univariate, multivariate analysis and nomogram constructed

**Figure 1:**
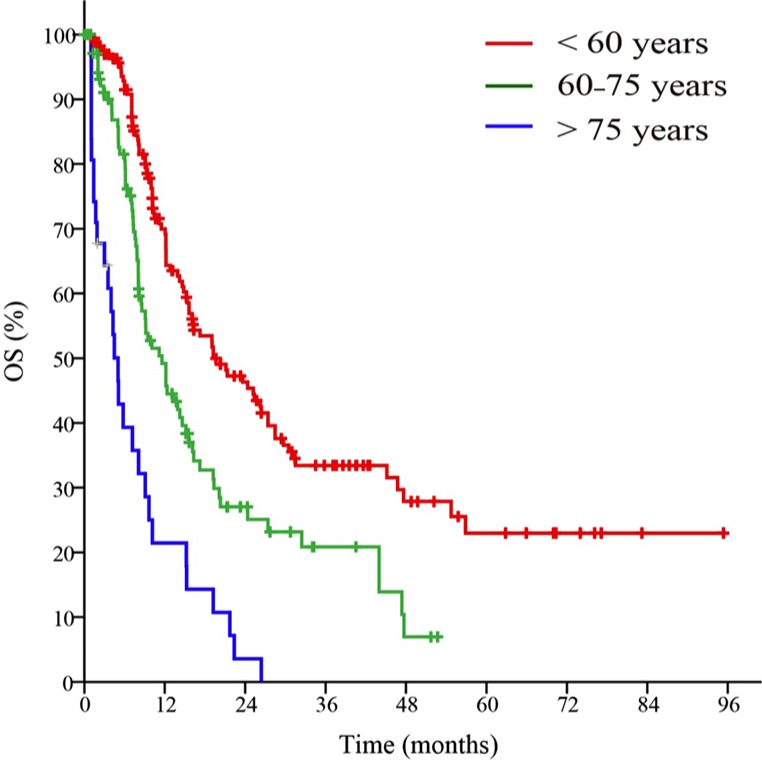
Survival curves of patients in the primary cohort based on the advanced age stratification

**Figure 2:**
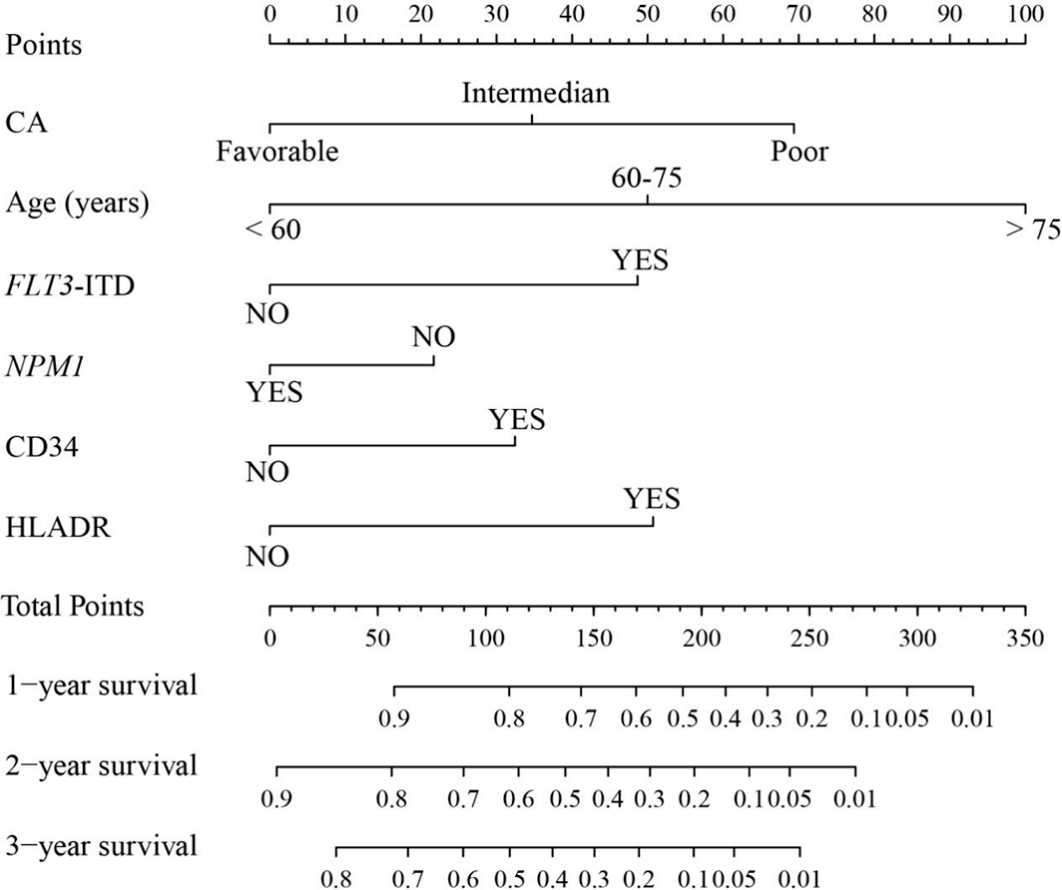
Nomogram for patients with acute myeloid leukemia Nomogram instructions: Each variable axis represents the value of every individual patient where located on. To determine the points of variable value, a line is drawn upwards to the points' axis. To determine the 1-year, 2-year and 3-year OS probability, a line is drawn downwards from the total points' axis where the total points summed up by points of each variable located on to the survival one. CA, cytogenetic abnormalities; *FLT3*-ITD, fms-related tyrosine kinase 3 internal tandem duplications; *NPM1*, nucleophosmin 1; HLA-DR, human leukocyte antigen DR; OS, overall survival.

The poor prognostic factors of OS were identified in 2 cohorts were as follows: age ≥ 60 years; stratified as poor risk of cytogenetic abnormalities; presence of *FLT3*-ITD mutation; absence of *NPM1* mutation; positive for CD34; positive for CD56; positive for HLA-DR in the primary cohort and age ≥ 60 years; presence of *FLT3*-ITD mutation; positive for MPO; positive for CD34; positive for CD56 in the validation cohort in the univariate analysis, respectively (Table 2). The results for the advanced age stratification were similar as that of the conventional age stratification in the log-rank test (Figure 1). Multivariate analyses verified independent risk factors included age, risk stratification of cytogenetic abnormalities, status of *FLT3*-ITD mutation, expression of CD34 and expression of HLA-DR (Table 3). A nomogram that predicts 1-year, 2-year and 3-year OS was constructed based on the results from the univariate and multivariate Cox's regression analyses in the primary cohort (Figure 2). The advanced age stratifications and the other independent risk factors described above were included. Considering the favorable influence of OS, status of *NPM1* mutation was also included into the model.

**Table 2:** Overall survival in univariate analysis for all cohorts

Factors	Primary cohort			Validation cohort		
	HR	95% CI	*P*	HR	95% CI	*P*
Sex (men)	1.097	0.822–1.464	0.530	1.051	0.727–1.519	0.793
Age (≥ 60 yrs)	2.436	1.822–3.258	< 0.001	2.768	1.898–4.036	< 0.001
CA (poor)	2.000	1.439–2.780	< 0.001	1.419	0.933–2.160	0.102
*FLT3*-ITD (+)	1.717	1.283–2.298	< 0.001	1.520	1.013–2.151	0.043
*NPM1* (+)	0.601	0.441–0.819	0.001	0.947	0.692–1.575	0.812
MPO (+)	0.708	0.486–1.031	0.072	0.607	0.384–0.959	0.033
CD34 (+)	1.944	1.345–2.809	< 0.001	1.279	1.098–1.770	0.039
CD56 (+)	1.906	1.390–2.614	< 0.001	1.245	1.178–1.706	0.041
HLA-DR (+)	3.514	1.930–6.400	< 0.001	2.064	0.961–3.532	0.106

Abbreviations: CA, cytogenetic abnormalities; *FLT3*-ITD, *fms-related tyrosine kinase 3* internal tandem duplications; *NPM1, nucleophosmin 1*; MPO, myeloperoxidase; HLA-DR, human leukocyte antigen DR; HR hazard ratio; OS, overall survival; CI, confidence interval.

**Table 3:** Multivariate analysis of patients in all cohorts

Factors	Primary cohort			Validation cohort		
	HR	95%CI	*P*	HR	95% CI	*P*
Age (≥ 60 yrs)	1.995	1.595–2.494	< 0.001	2.853	1.940–4.196	< 0.001
CA (poor)	1.806	1.291–2.526	0.001	−	−	−
*FLT3*-ITD (+)	2.029	1.492–2.759	< 0.001	1.656	1.070–2.560	0.023
*NPM1* (+)	0.808	0.585–1.117	0.197	−	−	−
CD34 (+)	1.649	1.104–2.462	0.015	1.198	0.887–1.564	0.107
CD56 (+)	1.377	0.990–1.915	0.057	1.102	0.732–1.589	0.694
HLA-DR (+)	1.919	1.013–3.637	0.046	−	−	−
MPO (+)	−	−	−	0.665	0.419–1.057	0.085

Abbreviations: CA, cytogenetic abnormalities; *FLT3*-ITD, *fms-related tyrosine kinase 3* internal tandem duplications; *NPM1, nucleophosmin 1*; HLA-DR, human leukocyte antigen DR; HR hazard ratio; OS, overall survival; CI, confidence interval.

### Nomogram validation

**Figure 3:**
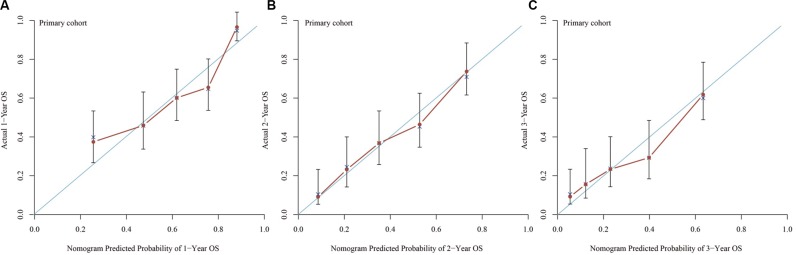
Calibration of internal validation: the calibration curve for the prediction of 1-year OS (A), 2-year OS (B) and 3-year OS (C); the predicted and the actual probability is plotted on the x and y axis, respectively

**Figure 4:**
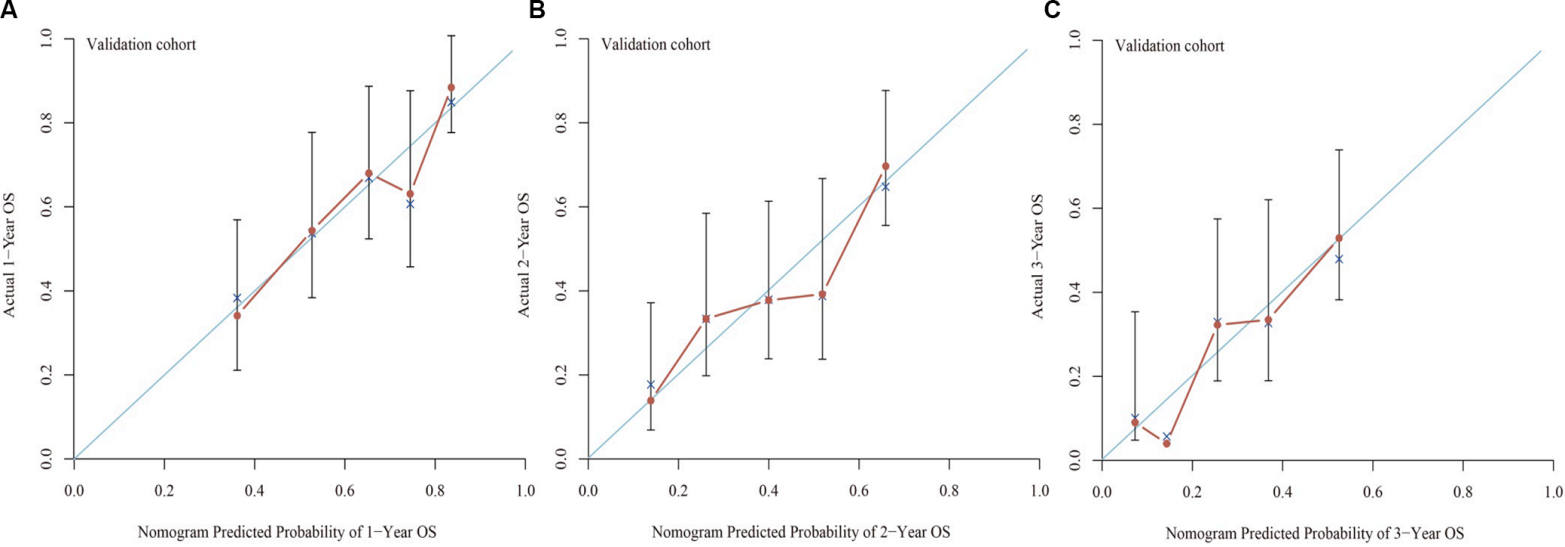
Calibration of external validation: the calibration curve for the prediction of 1-year OS (A), 2-year OS (B) and 3-year OS (C); the predicted and the actual probability is plotted on the x and y axis, respectively

The nomogram was externally validated in the validation cohort that contains 165 patients by calculating the bootstrap C statistics with the calibration plot. The predictive accuracy for 1-year, 2-year and 3-year of OS as examined by the concordance index (C-index) were 0.71 in the internal validation and 0.68 in the external validation, respectively. The value indicated that the model was equipped with comparatively accurate ability of discrimination. The calibration plot for the probability of 1-year (Figure 3A), 2-year (Figure 3B) and 3-year (Figure 3C) of OS indicated favorable correlation between the predictive outcome and actual observation in the internal validation. In the external validation, the 1-year (Figure 4A), 2-year (Figure 4B) and 3-year (Figure 4C) OS also demonstrated an optimistic concordance between them, which suggested that the nomogram was well calibrated.

### Contrast of the predictive ability between the nomogram and the current prognostic systems

**Figure 5:**
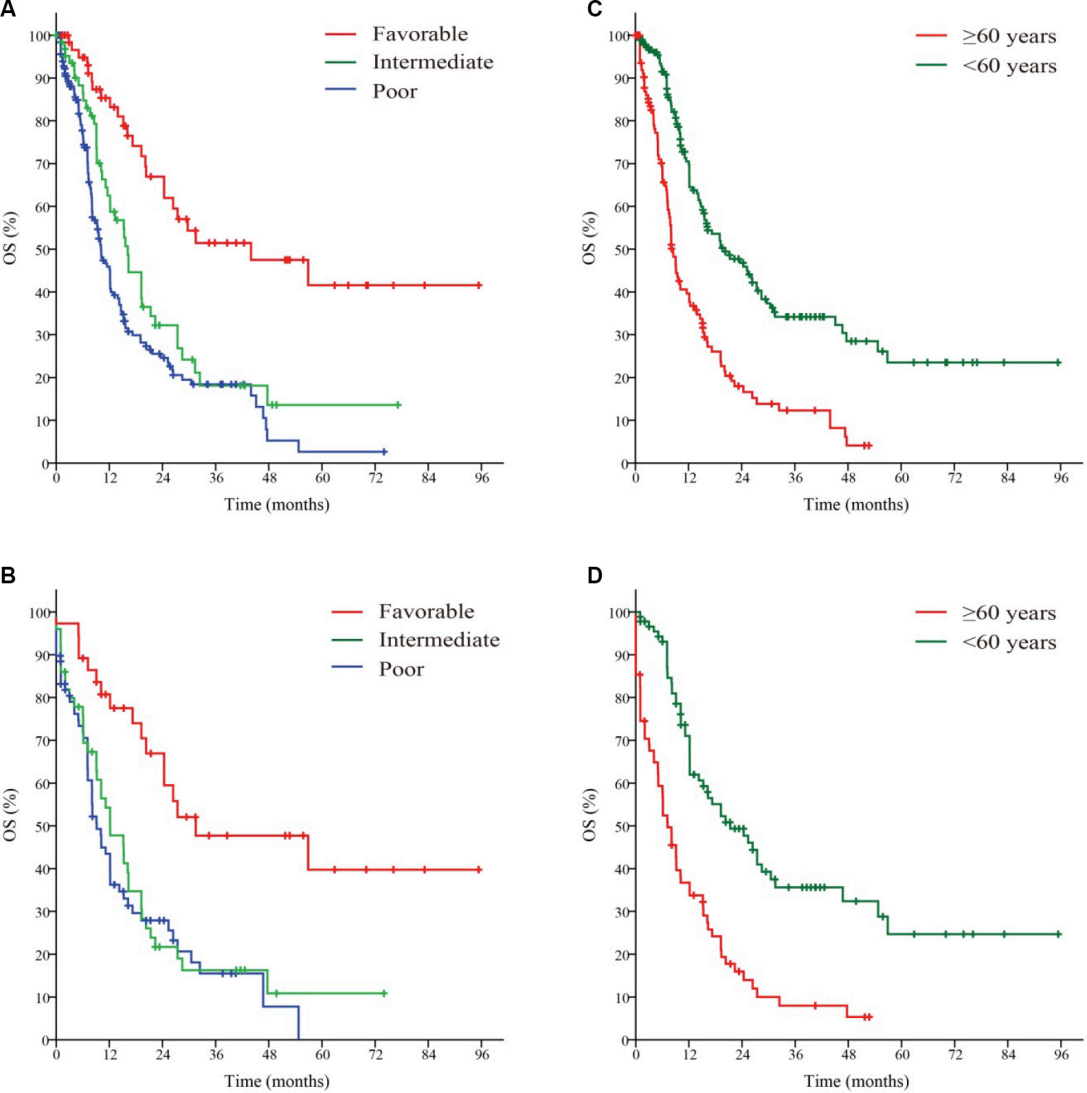
Survival curves of the primary cohort based on the Risk status (A), Age (C); and the validation cohort according to Risk status (B), Age (D) Risk status, the stratification of risk status based on cytogenetic and molecular abnormalities; Age, the conventional age stratification. The stratification of risk status based on cytogenetic and molecular abnormalities and the conventional age stratification are according to National Comprehensive Cancer Network (NCCN) Clinical Practice Guidelines in Oncology: Acute Myeloid Leukemia, Version 2009–2015.

**Figure 6:**
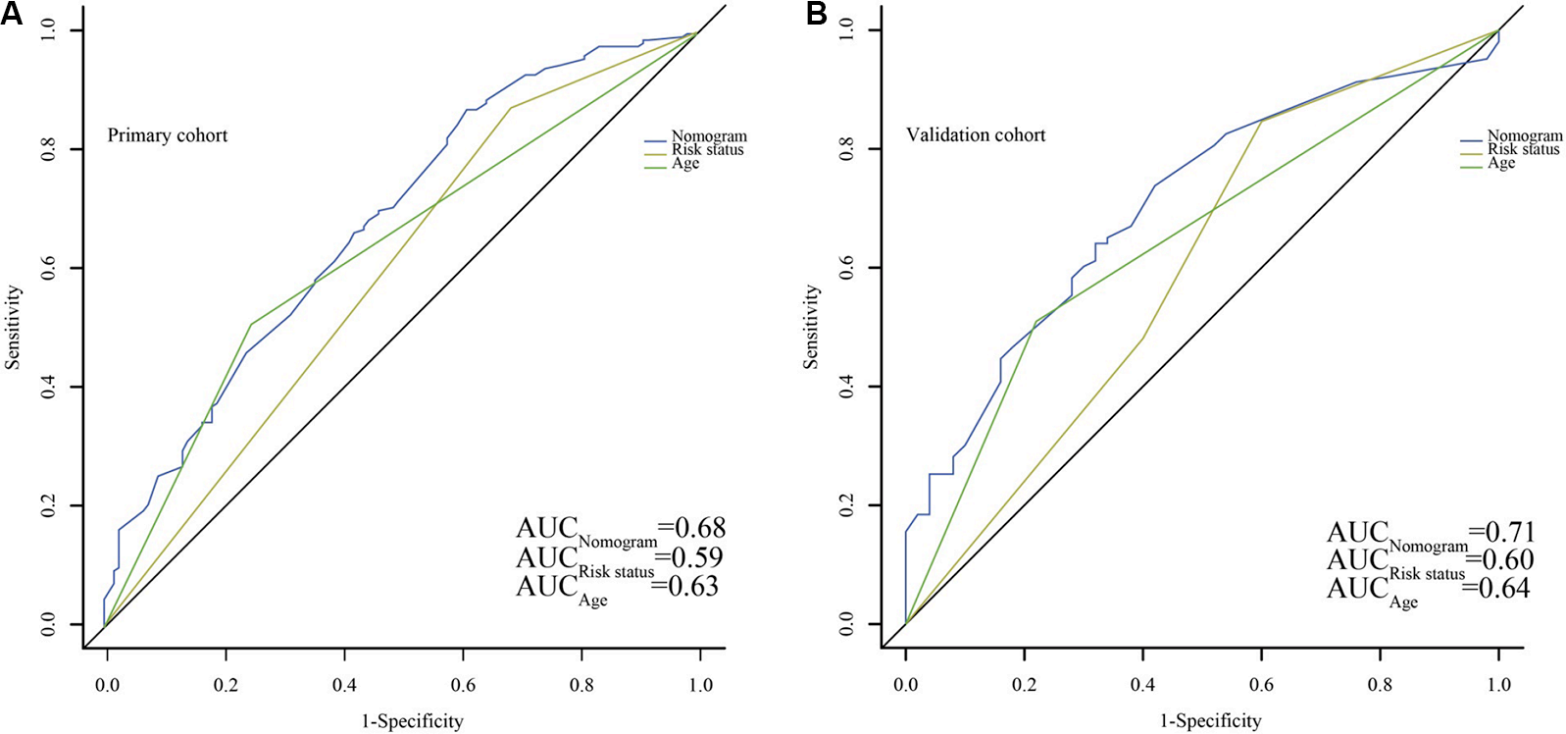
Comparison of the predict ability for overall survival (OS) between the nomogram and current prognostic systems in all patients Discrimination: The AUC of nomogram, Risk status and Age are 0.68, 0.59 and 0.63, respectively (**A**) in the primary cohort, while in the validation cohort, the AUC are 0.71, 0.60 and 0.64, respectively (**B**). Risk status, the stratification of risk status based on cytogenetic and molecular abnormalities; Age, the conventional age stratification. The stratification of risk status based on cytogenetic and molecular abnormalities and the conventional age stratification are according to National Comprehensive Cancer Network (NCCN) Clinical Practice Guidelines in Oncology: Acute Myeloid Leukemia, Version 2009–2015.

The risk stratification based on cytogenetic and molecular abnormalities and age were considered as independent prognostic factors. Except for poor risk based on validated cytogenetic and molecular abnormalities, age ≥ 60 years was stratified as poor risk in the NCCN Clinical Practice Guidelines in Oncology: Acute Myeloid Leukemia (Version 2009–2015). As shown in Figure 5, patients stratified as favorable risk based on validated cytogenetic and molecular abnormalities had an obviously favorable prognosis in both cohorts. However, it was not good enough for stratification of patients with intermediate and poor risks (Figure 5A and 5B). The conventional age stratification showed good ability of prognostic stratification in both cohorts (Figure 5C and 5D). The nomogram was more accurate for predicting OS in both cohorts when compared with the prognostic stratifications mentioned before. The C-index of that in the primary cohort (0.71) was better than the cytogenetic and molecular abnormalities stratification (0.62), and conventional age stratification (0.63). The Similar result was observed in the validation cohort as the C-index of the cytogenetic and molecular abnormalities stratification (0.60) and conventional age stratification (0.62) were lower than that of the nomogram (0.68). The area under the receiver operating characteristic (ROC) curve (AUC) of nomogram (0.68) was higher than that of risk stratification based on validated cytogenetic and molecular abnormalities (0.59) and conventional age stratification (0.63) in the primary cohort (Figure 6A). Similar result was observed in the validation cohort. The AUC were 0.71, 0.60 and 0.64, respectively (Figure 6B). All results indicated that this nomogram was more precise and reliable for the OS prediction in adult patients with AML.

## DISCUSSION

The diversity and heterogeneity of non-APL AML have determined that it may take detours to approach the truth of creating an appropriate predictive model to give an indication of prognosis. The efforts have been made by groups, and variable results are expected [12–15]. The clinical evaluation variables incorporated into the nomogram are strongly recommended by NCCN Clinical Practice Guidelines in Oncology: Acute Myeloid Leukemia, and will be recommended and documented by most hematologist, which means it is practical for any medical institutions without any serious restriction. The nomogram has been developed with AML patients at one Chinese institution and validated with patients from TCGA. Based on a certain number of heterogenous patients, the nomogram is improved for some of the current independent prognostic factors. Over the last decade, more and more targets have emerged as significant prognostic factors [16–19]. Most of the clinical variables we utilized have been previously associated with OS. The other variables such as HLA-DR is easy to be ignored by physicians and prognostic factors are considered to be potentially correlated based on some studies [20, 21].

In our nomogram, the most significant factor regarded to prognostic correlation in the Cox's regression analysis was risk stratification based on cytogenetic abnormalities and age, which was concordant with the NCCN Clinical Practice Guidelines in Oncology: Acute Myeloid Leukemia. We also observed that positive HLA-DR had an adverse effect on OS in all cohorts, which had not been reported previously. Hence, we believed that positive HLA-DR may be a novel prognostic factor. Because we sought to create a predictive utility for OS before treatment, different treatment regimens were excluded in the survival analyses. Furthermore, therapeutic regimens of patients in this study were generally different. Therapeutic regimens vary for they are planned according to different stratification systems of NCCN Clinical Practice Guidelines in Oncology: Acute Myeloid Leukemia, such as age stratification (< 60 or ≥ 60 years old) as well as cytogenetic and molecular abnormalities risk stratification. However, the regimen for patients with the same system in our study is of consistency. In order to achieve the consistency of therapy baseline, our models include these two systems and the age and risk factor are scored prior to other factors. Hence, their OS can be comparable. Also further studies should aim to establish a more detailed risk-adapted therapy for stratified patients.

A nomogram integrates different clinical variables to provide an individualized prognosis assessment, which shows more benefits contrasted to current prognostic systems in several clinical settings [22–24]. In accordance with previous studies, our nomogram made a more favorable and detailed prediction of OS than the current prognostic systems. The C-index were 0.71 and 0.68 in the primary and validation cohort respectively, while the C-index of the cytogenetic and molecular abnormalities risk stratification and conventional age stratification were all ≤ 0.63 (range, 0.60–0.63). For the AUC, the results were consistent with the C-index. The risk stratification based on cytogenetic and molecular abnormalities assigned patients to 3 subgroups with relatively distinctive predicted survivals, but it was not so effective to separate patients into different risk groups in our study. A convergence between intermediate risk and favorable risk was observed on the Kaplan–Meier curves for OS in both cohorts. The conventional age stratification was clearly satisfactory in segregating patients with predicted survivals, but it was no superior to the nomogram.

Satish Krishnan *et al*. [25] have demonstrated an effective prognostic nomogram for elderly AML patients treated with intensive chemotherapy in 2011 American Society of Hematology annual meeting abstract previously. However their variables and prognostic factors included are quite different from this study. To the best of our knowledge, there have not been any other nomograms concerning clinical outcomes of patients with AML published yet.

There are several limitations to our nomogram. Some convincing variables, such as *c-KIT*, *CEBPA* mutations, prothrombin time, fibrinogen, and CT/MRI if neurologic symptoms present, were not included [26–30]. However, analyses for many independent prognostic molecular markers are not widespreadly available. Our nomogram was principally constructed based on limited area and patient population, and it is uncertain whether the nomogram is precise for patients from the other regions, or larger populations of patients from other areas of China.

In summary, we performed a nomogram that can predict 1-year, 2-year and 3-year OS for AML based on a certain amount of patients with multi-subtype disease and heterogenous treatments, and it was validated with an appropriate level of accuracy. It is better in stratification than the current prognostic system, and can help both patients and physicians to make prediction of OS individually prior to the treatments.

## MATERIALS AND METHODS

### Patients

The primary cohort comprised 311 hospitalized patients with previously untreated non-APL AML. The patients were recruited from the Department of Hematology and Oncology of our institution between September 2009 and June 2015, and were diagnosed based on typical morphology, immunophenotype, cytogenetic and molecular makers according to the World Health Organization classification. Patients were excluded if they lost to follow-up. For validation, we used publically available data of patients with AML generated with Cancer Genome Atlas Research Network (TCGA). The clinical data of level 1–3 were downloaded from TCGA (http://tcga-data.nci.nih.gov/tcga/). The validation cohort consisted of 165 patients. The study protocol was performed in accordance with the guidelines outlined in the Declaration of Helsinki and was approved by the Ethics Committee of Third Affiliated Hospital of Soochow University.

### Evaluation and treatment

Previous evaluations included a history and physical examination, differential blood count, bone marrow with cytogenetics (karyotype ± fluorescence *in situ* hybridization) and molecular analyses (*FLT3*-ITD and *NPM1* mutations) and immunophenotype. Cytogenetic abnormalities were stratified according to NCCN Clinical Practice Guidelines in Oncology: Acute Myeloid Leukemia (Version 2009–2015).

Contrast to the conventional age stratification (age < 60 years and age ≥ 60 years), we performed advanced stratification methods based on age, which stratified the age of the patients as < 60 years, 60–75 years and > 75 years.

All the patients received treatments rigorously recommended by NCCN Clinical Practice Guidelines in Oncology: Acute Myeloid Leukemia after diagnoses. All the patients tolerated to anthracycline. The younger patients (age < 60 years) received standard-dose cytarabine at 150 mg/m^2^ continuous infusion × 7 days with idarubicin at 12 mg/m^2^ or daunorubicin 60 mg/m^2^ × 3 days as induction treatment, while the patients aged between 60 and 75 years received standard-dose cytarabine at 100 mg/m^2^ infusion × 7 days with reduced-dose idarubicin at 8 mg/m^2^ or daunorubicin at 45 mg/m^2^, and elder patients (age > 75 years) received low-dose cytarabine at 10 mg/m^2^ hypodermic injection ×14 days with harringtonine at 1mg/m^2^ infusion and G-CSF 300 μg/d. Then, the same regimens were given as post-induction therapy for all patients. High-dose cytarabine (HiDAC) at 3 g/m^2^ every 12 h on days 1, 3, 5 for 3–4 cycles were conducted as consolidation therapy in younger patients who achieved complete response (CR), and HiDAC at 2 g/m^2^ every 12 hours × 6 days with idarubicin at 12 mg/m^2^ or daunorubicin at 60 mg/m^2^ × 3 days were given to those with failure to induction treatment as the re-induction treatment. For older patients with CR, 3–4 cycles of consolidation therapy were given as the same as the post-induction therapy. No more aggressive therapy but qualified supportive care was given to the older patients with failure induction treatment. There were 33 younger patients received hematopoietic stem cell transplantation (HSCT) after achieving CR, among whom 12 were autologous-HSCT and 21 were matched sibling or alternative donor-HSCT. Of these 33 patients, 20 patients died in 3 years due to primary disease, graft-versus-host disease or infection.

### Design and validation of the nomogram

Several clinical features correlated with OS were identified and integrate as prognostic variables. Some variables have been demonstrated previously [31]. The prognostic factors included sex, age and risk stratifications based on cytogenetic abnormalities, status of *FLT3*-ITD mutation, status of *NPM1* mutation, expression of CD34, expression of CD56 and expression of HLA-DR. Multivariate Cox proportional hazard model was applied. Validation was performed at stages listed below. Internal validation was conducted with C-index, which was computing based on the AUC. The maximum value of the c-index was 1.0, indicating a perfect discrimination, whereas 0.5 indicated a random chance to correctly discriminate outcome with the model [32]. Then, bootstrap resampling (1,000 resamples) was used for a calibration plot, which was constructed to verify the concordance between the predicted and observed probabilities. Finally, external validation was performed. Patients of validation cohort were assessed by the nomogram. The total score of every patient was included in further Cox regression analysis as independent factors. The C-index was then derived from analysis above, and the calibration curve was drawn.

### Statistical analyses

The Statistical Package for the Social Sciences (SPSS) software version 19 (SPSS Inc., Chicago, IL, USA) and the foreign, rms, pROC package in R, version 3.2.2 (http://www.r-project.org/) were applied for statistical analysis. OS was estimated from the time when diagnosed to the time of death. The descriptive statistics were reported as the proportion. Kaplan-Meier (Log-rank test) and Cox's regression model were used for univariate and multivariate survival analysis, respectively. The nomogram was built by the Cox's model parameter calculating of the primary cohort. A backward step down-selection process was applied for the final model selection. The construction and validation of nomogram were conducted according to Iasonos' guide [33]. To measure model discrimination of nomogram, AUC which ranged from 0.5 to 1.0 (best) was used. The difference was considered statistically significant when *P* < 0.05. The codes of R project are listed below for reference (non-unique).

**Design**

ibrary(foreign)

bc<-read.spss(‘**filename**’,use.value.labels=T,to.data.frame=T)

library(rms)

coxm<-cph(Surv(OS,status,type=”right”)~**variable1**+**variable2**+**variable3**+…+ **variableN**,x=T,y=T,data=bc,surv=T)

scoxm<-step(coxm)

dd<-datadist(bc)

options(datadist=”dd”)

surv<-Survival(coxm)

surv1<-function(x) surv(1*12,lp=x)

surv2<-function(x) surv(2*12,lp=x)

surv3<-function(x) surv(3*12,lp=x)

nom<-nomogram(coxm,fun=list(surv1,surv2,surv3),lp=F,funlabel=c(‘1-year survival’, ‘2-year survival ‘, ‘3 year survival’),maxscale=100,fun.at=c(0.9,0.8,0.7,0.6,0.5,0.4,0.3,0.2,0.1,0.05,0.01))

plot(nom)

survConcordance(formula=Surv(OS,status)~predict(coxm),data=bc)

**Validation**

library(foreign)

bc<-read.spss(‘**filename**’,use.value.labels=T,to.data.frame=T)

library(rms)

coxm<-cph(Surv(OS,status,type=”right”)~ **variable1**+**variable2**+**variable3**+…+ **variableN**,x=TRUE,y=TRUE, data=bc,surv=TRUE,time.inc=**n**)

dd<-datadist(bc)

options(datadist=”dd”)

cal <-calibrate(coxm, cmethod='KM’, method=”boot”,u=**n**,m=**x**, B=1000)

par(mar = c(8,5,3,2),cex = 1.0)

plot(cal,lwd=1,lty=1,errbar.col=c(rgb(0,0,0,maxColorValue = 255)),xlim = c(0,1),ylim= c(0,1),xlab=”Nomogram Predicted Probability of N-Year OS”,ylab=”Actual **N**-Year OS”,col=c(rgb(255,0,0,maxColorValue =255)))

lines(cal[,c(‘mean.predicted’,'KM’)], type = ‘b’,lwd =2, col = c(rgb(192,98,83,maxColorValue = 255)),pch = 16)
